# Increased cesarean section rate and premature birth according to modified WHO maternal cardiovascular risk in pregnant women with congenital heart disease

**DOI:** 10.1371/journal.pone.0294323

**Published:** 2023-11-16

**Authors:** Frida Wedlund, Emma von Wowern, Joanna Hlebowicz

**Affiliations:** 1 Department of Cardiology, Skåne University Hospital, Lund University, Malmö, Sweden; 2 Clinical Sciences, Lund University, Lund, Sweden; 3 Institution of Clinical Sciences Malmö, Perinatal and Cardiovascular Epidemiology, Lund University, Lund, Sweden; 4 Dept. of Obstetrics and Gynecology, Skåne University Hospital, Malmö, Sweden; 5 Department of Cardiology, Skåne University Hospital, Lund University, Lund, Sweden; University of Mississippi Medical Center, UNITED STATES

## Abstract

**Background:**

During pregnancy and delivery, hemodynamics are altered and complex congenital heart disease has been associated with adverse maternal and neonatal outcomes. We sought to investigate pregnancy outcome and complications in relation to complexity of heart condition.

**Materials and methods:**

We studied women with ACHD discussed at multidisciplinary conferences at Lund University Hospital March 2009-May 2021. We studied 149 pregnancies in 101 women. We scored each woman retrospectively according to the modified World Health Organization (mWHO) risk classification and included patients in risk class I (n = 36, 24.1%), II (n = 43, 28.9%), II-III (n = 43, 28.9%), III (n = 24, 16.1%) and IV (n = 3, 2.0%).

**Results:**

Women with mWHO class ≥III underwent cesarean section more often than women in less complex mWHO classes, (OR, 5.1; 95% CI, 2.0–12.5; *p*<0.001). The odds of premature delivery were significantly higher among pregnant women with mWHO class ≥III (OR, 6.7; 95% CI, 2.6–17.4; *p*<0.001). We found no difference in incidence of preeclampsia, gestational hypertension, gestational diabetes, hemorrhage >1000 ml or cardiac defect in the neonate depending on WHO-class. Women in mWHO classes III-IV had a higher rate of fetal growth restriction (FGR) compared to women in mWHO classes I, II, II-III (p<0.007).

**Conclusions:**

Our findings indicate that women with more complex heart disease (mWHO classes III or IV) tend to have a higher rate of cesarean section, premature birth and FGR.

## Introduction

Health care for children born with congenital heart disease have improved and over 97% reach adulthood [1]. In Sweden, we have approximately 40,000 patients with Adult Congenital Heart Disease (ACHD). As of 2020, around 5000 women are under 50 years of age, according to the Swedish Registry of Congenital Heart Disease (SWEDCON) [2]. Pregnancy and delivery are associated with considerable hemodynamic changes, even in healthy women [3]. In 2018, the World Health Organization published recommendations on how to classify women’s cardiovascular risk: the modified World Health Organization (mWHO) risk classification score. The woman is classified into mWHO risk classes I-IV, depending on the type of congenital heart disease and functional status. According to guidelines from The European Society of Cardiology, the mWHO risk classification score should be used in women with ACHD during pregnancy [4]. This risk classification is also useful in women with ACHD for prediction of cardiac events during pregnancy [5]. During pregnancy, it is recommended that women with more than moderate-to-complex ACHD are the subject of multidisciplinary discussions, with involvement from the anesthesiologist, obstetricians and ACHD cardiologists [6].

Women with complex congenital heart disease has been associated with adverse maternal and neonatal outcomes. The incidence of giving birth to a small for gestational age (SGA) neonate or giving birth preterm is increased, especially in women with more complex ACHD [7–9]. Another complication that might occur more frequently is fetal growth restriction (FGR); particularly described in women suffering from cyanotic heart condition without pulmonary hypertension (oxygen saturation<85%), unrepaired atrial septal defect (ASD), Tetralogy of Fallot and valvular conditions (mitral stenosis, valvular aortic stenosis and regurgitant lesions). Hypertensive disorders and certain medications, such as betablockers, are also associated with a higher incidence of FGR [4]. Moreover, women with ACHD have an increased risk of obstetric complications such as preeclampsia and postpartum hemorrhage [6].

Pregnancy in women with congenital heart disease is an important research area; more and more women with ACHD are choosing to have a baby [10] and nowadays women with more complex ACHD have given birth [11]. This is despite the latest WHO recommendations that pregnancy is contraindicated in such women and, if pregnancy occurs, termination should be discussed, as detailed in mWHO IV [4]. At our tertiary center in southern Sweden we treat women with ACHD during pregnancy and delivery. As described above, complex congenital heart disease has been associated with adverse maternal and neonatal outcomes. Thus, we sought to investigate perinatal outcome in our cohort in order to better stratify for risk and improve preconceptional counselling as well as pregnancy management in ACHD women in this setting. The management of women with ACHD during pregnancy and childbirth can differ both nationally and internationally making it important to study our cohort of this heterogenous group of heart diseases.

## Materials and methods

**Fig 1:**
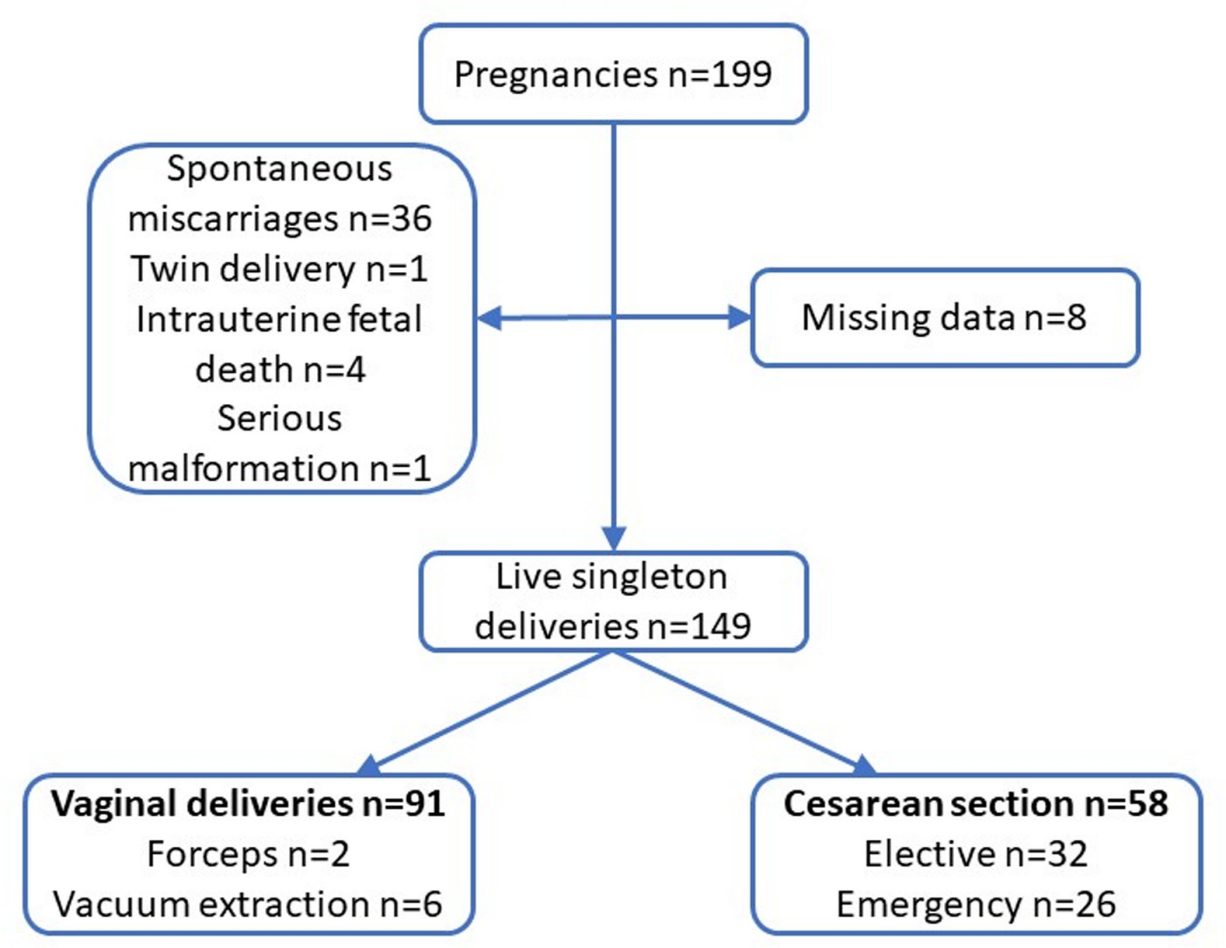
Pregnancy outcome and delivery mode in women with adult congenital heart disease.

As a routine procedure, the cases of all pregnant women with ACHD are discussed at a multidisciplinary conference to plan the pregnancy and delivery. All consecutive pregnant patients with ACHD at the tertiary ACHD Center at Skåne University Hospital (Lund, Sweden) whose cases were discussed March 2009-May 2021 were included. Women giving birth outside of Skåne County (n = 8), twin pregnancies (n = 1), serious malformations (n = 1), spontaneous miscarriages (n = 36), intrauterine fetal deaths > 22 weeks of gestation (n = 4) and induced abortions were excluded from analysis (Fig 1).

Baseline data were collected from the SWEDCON registry, medical and obstetrical records. Women undergoing surgery for atrial septal defect (ASD) before reaching 18 years were classified as “operated”, otherwise the women were classified as “nonoperated”. We classified women with an ASD according to presence of surgery before 18 years of age. Due to the small number of patients in each diagnosis group, outcome analyses were performed according to mWHO class and not anatomical diagnosis.

Neonatal/fetal complications were defined:

1. Preterm birth (<37 weeks of gestation), regardless of iatrogenic or spontaneous delivery;
2. Small for gestational age (SGA), birth weight below two standard deviations from the mean weight corrected for gender and gestational week;
3. Cardiac birth defects: ICD-10 Q code 20–26;
4. Clinical diagnosis of FGR as noted in the medical records. FGR is diagnosed clinically when the estimated fetal weight is <-22% compared to average according to Swedish standard in combination with abnormal umbilical artery Doppler measurements, and/or the fetal growth between two ultrasound measurements 2 weeks apart declines >10% over 2 weeks;

Pregnancies were scored retrospectively using the mWHO risk classification, the first clinical visit and the first echocardiography during pregnancy [4]. The following assessments were made: transposition with previous arterial switch procedure and without aortic dilatation: mWHO II, transposition with previous Mustard/Senning procedure: mWHO III; severe aortic regurgitation: mWHO II-III, severe symptomatic pulmonary stenosis with mildly decreased right ventricle function: mWHO III, well-functioning Ross: mWHO I and well-functioning corrected anomalous left coronary artery from the pulmonary artery: mWHO II.

We collected ultrasound data and defined blood flow class (BFC) 1 as positive diastolic flow and +2 SD > pulsatility index (PI) ≤ +3 SD, BFC 2 as positive diastolic flow and PI > +3 SD, BFC 3A as absence of diastolic blood flow and BFC 3B as negative diastolic blood flow in the umbilical artery. Uterine artery score is a locally used classification quantifying uterine artery blood flow resistance from normal (0) to a maximum of 4 indicating PI > 1.2 and/or presence of end-diastolic notch in the uterine arteries bilaterally.

### Statistical analyses

The continuous variables body mass index, age, length of gestation, pH umbilical artery and vein were tested for normal distribution with the Shapiro-Wilk test. The distribution of body mass index and pH umbilical vein were found to be skewed and were log transformed to calculate the z-score. Normally distributed continuous variables are presented as mean±SD or proportions. Non-normally distributed variables were log transformed for analysis and are presented in non-logarithmic format as median and interquartile range.

Fisher’s exact test was used to test for statistical significance between categorical variables and mWHO groups. Independent sample Kruskal-Wallis test was used to test for statistical significance between continuous variables and mWHO groups. Pearson’s Chi-Square test was used to test for statistical significance correlation between cardiac birth defects in the neonate and diagnosis of diabetes or gestational diabetes in the ACHD women and prematurity <37 weeks, weeks 28–32 and <28 weeks in relation to iatrogenic versus spontaneous childbirth.

Cesarean delivery rates and mWHO classes over time were examined using the Cochran-Armitage χ2.

Pearson’s Chi-Square test was used to test for statistical significance correlation between mWHO class, delivery mode, age, the use of betablockers, blood thinners and previous cesarean section. Due to the small number of women in mWHO class IV we compared mWHO classes I, II, II-III (mWHO group I-III) with mWHO classes III and IV (mWHO group III-IV). We estimated the odds ratio with 95% confidence intervals as the measure of the association between premature delivery or cesarean section and mWHO class. Univariate logistic regression analysis was used to examine the association between prematurity and mWHO class, respectively cesarean section and mWHO-class. Then we performed a stepwise multivariate regression analysis adjusting for age and either BMI, the use of betablockers, blood thinners (acetylsalicylic acid, low molecular weight heparin, P2Y12-inhibitors) or previous cesarean sections. We also performed a sensitivity analysis where we specifically examined nulliparous women with ACHD to exclude the impact of a previous cesarean section or premature delivery affecting the probability of another adverse event. The level of significance was set to *p* < 0.05. We used IBM Corp. Released 2020, IBM SPSS Statistics for Windows, Version 27.0 (Armonk, NY: IBM Corp) for statistical calculations.

### Ethical approval

This study was approved by the Ethics Committee for Human Research 2021-05-17 reference number 2021–01636. The need for consent was waived by the ethics committee.

## Results and discussion

### Demography and cohort characteristics

The subsequent study cohort included 149 pregnancies in 101 women with ACHD “Table 1”. In two of the pregnancies the women were diagnosed with type 1 diabetes before the pregnancy, and in 11 pregnancies the women were diagnosed with gestational diabetes. In two non-diabetic pregnancies, the women had a mechanical valve.

**Table 1:** Clinical characteristics. Data are shown as numbers and as percentage. Skåne University Hospital, SUS, Lund is the tertiary center located in Skåne County. BMI was measured at the first visit at the prenatal care unit. Regarding medications, the patient was considered to be taking a prescribed medication during pregnancy even if the medication was only prescribed for part of the pregnancy. All women with missing data during pregnancy were considered not to have been prescribed any medications, if the report in medical journals before or after pregnancy indicated that they did not take any medications. Blood thinners is defined as the use of acetylsalicylic acid, P2Y12-inhibitors or low molecular weight heparin.

	mWHO I (%) n = 36 (24.1%)	mWHO II (%) n = 43 (28.9%)	mWHO II-III (%) n = 43 (28.9%)	mWHO III (%) n = 24 (16.1%)	mWHO IV (%) n = 3 (2.0%)	Missing n (%)
Age (years) *(p = 0.442) Mean ± SD BMI (kg/m^2^) *(p = 0.028) Median IQR Parity Parity 0 Parity 1 Parity ≥2 Diagnoses DORV Transposition ‐ Mustard/Senning Transposition ‐ arterial switch Aortic anomalies Fallot’s anomaly Aortic valve lesions Mitral valve lesions Pulmonary valve lesions Shunt lesions op<18 years Shunt lesions not op<18 years Single ventricle Coronary anomalies	32 ± 5 22.9 21.87–24.9 19 (52.8) 14 (38.9) 3 (8.3) 0 0 0 0 0 9 (25.0) 10 (27.8) 2 (5.6) 11 (30.6) 4 (11.1) 0 0	30 ± 5 24.2 22.36–28.7 19 (44.2) 17 (39.5) 7 (16.3) 6 (14.0) 0 5 (11.6) 0 17 (39.5) 3 (7.0) 0 0 2 (4.7) 9 (20.9) 0 1 (2.3)	31 ±4 22.6 20.8–24.8 21 (48.8) 18 (41.9) 4 (9.3) 0 0 0 18 (41.9) 3 (7.0) 19 (44.2) 1 (2.3) 0 2 (4.7) 0 0 0	31 ± 6 24.5 19.7–27.7 11 (45.8) 8 (33.3) 5 (20.8) 0 3 (12.5) 1 (4.2) 2 (8.3) 0 4 (16.7) 6 (25.0) 2 (8.3) 0 0 5 (20.8) 1 (4.2)	31 ± 3 22.2 2 (66.7) 0 1 (33.3) 0 0 0 1 (33.3) 0 1 (33.3) 1 (33.3) 0 0 0 0 0	0 8 0 0
Smoking No 1–9 cigarettes/day >10 cigarettes/day Snuff	33 (91.7) 0 2 (5.6) 0	30 (69.8) 7 (16.3) 4 (9.3) 0	38 (88.4) 1 (2.3) 0 0	14 (58.3) 0 3 (12.5) 0	3 (100) 0 0 0	14 (9.4)
ECG before Sinus rhythm Pacemaker rhythm Bradycardia	21 (58.3) 1 (2.8) 0	24 (55.8) 1 (2.3) 0	28 (65.1) 0 0	16 (66.7) 0 1 (4.2)	1 (33.3) 0 0	56 (37.6)
ECG during Sinus rhythm Pacemaker rhythm	19 (100) 0	26 (96.3) 1 (3.7)	36 (100) 0	22 (100) 0	3 (100) 0	42 (28.1)
Previous delivery Vaginal Cesarean section Both VBAC	11 (30.6) 6 (16.7) 0 2 (5.6)	19 (44.2) 5 (11.6) 0 0	15 (34.9) 7 (16.3) 0 1 (2.3)	6 (25.0) 6 (25.0) 1 (4.2) 2 (8.3)	0 1 (33.3) 0 0	0
Tertiary center Lund Medications Betablockade (new) Betablockade (continued) Betablockade (any) *(p<0.001) Acetylsalicylic acid (new) Acetylsalicylic acid (continued) Low molecular heparin (new) P2Y12-inhibitors (continued) Blood thinner (any) *(p<0.001) Digitalis (new) Diuretics (new) Diuretics (continued) Labetalol (new) Flecainide (new) Calcium channel blocker (new) No Instrumental Vacuum extraction Forceps	30 (83.3) 0 1 (2.8) 1 (2.8) 0 1 (2.8) 1 (2.8) 0 2 (5.6) 0 0 1 (2.8) 0 0 0 33 (91.7) 3 (75.0) 1 (25.0)	24 (79.1) 3 (7.0) 3 (7.0) 6 (14.0) 2 (4.7) 5 (11.6) 3 (7.0) 1 (2.3) 8 (18.6) 1 (2.3) 1 (2.3) 0 1 (2.3) 1 (2.3) 1 (2.3) 30 (70.0) 1 (100) 0	29 (67.4) 1 (2.3) 3 (7.0) 4 (9.3) 0 0 1 (2.3) 0 1 (2.3) 0 0 0 0 0 0 38 (88.3) 1 (50.0) 1 (50.0)	20 (83.3) 3 (12.5) 1 (4.2) 4 (16.7) 1 (4.2) 7 (29.2) 6 (14.3) 0 9 (37.5) 1 (4.2) 1 (4.2) 0 1 (4.2) 0 1 (4.2) 11 (45.8) 1 (100) 0	3 (100) 3 (100) 0 3 (100) 0 0 0 0 0 (100) 0 1 (33.3) 0 0 0 0 0 0 0	0 0 0

DORV = Double outlet right ventricle; ECG = electrocardiogram; mWHO class = modified World Health Organization class. IQR = interquartile range, VBAC = vaginal birth after cesarean section.

Normally distributed continuous variables are presented as mean ± SD or proportions. Non-normally distributed variables are presented in non-logarithmic format as median and interquartile range (IQR).

*Independent sample Kruskal-Wallis test was used to test for statistical significance between continuous variables and mWHO groups.

### Fetal assessment during pregnancy

In 88 pregnancies (59.0%) the woman underwent one or several ultrasound scans with Doppler blood flow velocimetry. In four pregnancies there was a pathological umbilical artery blood flow. In three pregnancies the BFC was 1. In one pregnancy the BFC increased throughout pregnancy (maximum BFC 3A), and the woman gave birth preterm to a neonate with low birth weight. In 13 pregnancies the woman had increased resistance of blood flow in the uterine arteries measured with Doppler blood flow velocimetry.

In 138 (92.6%) of the pregnancies, the woman had undergone ultrasound to predict the fetal weight. 11 pregnancies had a predicted low birth weight deviating by ≥ 22% and eight of these births were preterm.

Five women (3.4%) were diagnosed with FGR. One FGR was observed of 122 women (0.8%) in mWHO classes I, II, II-III compared with 4 out of 27 (14.8%) in mWHO classes III and IV p<0.007.

### Obstetric outcome

In 122 women of mWHO group class I-III, 39 (32.0%) cesarean sections were observed compared to 19 (70.4%) cesarean sections among 27 women in mWHO class group III- IV, see “Table 2”.

**Table 2:** Maternal outcome.

	mWHO I (%) n = 36 (24.1%)	mWHO II (%) n = 43 (28.9%)	mWHO II-III (%) n = 43 (28.9%)	mWHO III (%) n = 24 (16.1%)	mWHO IV (%) n = 3 (2.0%)	Mis- sing (%)	*p*-value
Hypertensive disease HELLP syndrome Preeclampsia Gestational diabetes Gestational age at delivery (weeks) Mean ± SD Preterm delivery (before week 37+0 and 32+0) Preterm delivery (before week 32+0) Induction	0 0 2 (5.6) 3 (8.3) 39.6 ± 2.3 1 (2.8) 1 (2.8) 10 (27.8)	2 (4.7) 0 2 (4.7) 2 (4.7) 38.8 ± 2.1 5 (11.6) 1 (2.3) 11 (25.6)	1 (2.3) 1 (2.3) 2 (4.7) 2 (4.7) 38.7 ± 2.7 3 (7.0) 2 (4.7) 9 (20.9)	1 (4.2) 0 0 3 (12.5) 36.9 ± 2.7 8 (33.3) 2 (8.3) 3 (12.5)	0 0 0 1 (33.3) 36.2 ± 1.7 2 (66.7) 0 0	0 0 0 0 0 0 0 0	0.72 1.0 0.86 0.254 <0.001* <0.001 0.077 0.077
Cesarean section Cardiac indication for induction/elective CS	11 (30.6) 1 (5.3)	13 (30.2) 3 (13.0)	15 (34.9) 5 (22.7)	16 (66.7) 9 (52.9)	3 (100) 3 (100)	0 0	0.004 <0.001
Emergency cesarean section	7 (19.4)	5 (11.6)	7 (16.3)	6 (25.0)	1 (33.3)	0	0.016
Haemorrhage >1000 ml	2 (5.6)	4 (9.3)	3 (7.0)	1 (4.2)	0	0	0.952

Data are shown as numbers and percentage. mWHO class = modified World Health Organization class.

Normally distributed continuous variables are presented as mean ± SD or proportions. Non-normally distributed variables are presented in non-logarithmic format as median and interquartile range (IQR).

Fisher’s exact test was used to test for statistical significance between categorical variables and mWHO groups.

*Independent sample Kruskal-Wallis test was used to test for statistical significance between continuous variables and mWHO groups.

Correlations were observed between delivery mode and mWHO group (r = 0.303, p-value <0.001), the use of betablockers (r = 0.211, p = 0.01), blood thinners (r = 0.170, p = 0.038) and previous cesarean sections (r = 0.395, <0.001). Women with mWHO class ≥III underwent cesarean section more often than women in less complex mWHO classes, (OR, 5.1; 95% CI, 2.0–12.5; *p*<0.001). Then we performed a stepwise multivariate logistic regression analysis adjusting for age and either, BMI, the use of betablockers, blood thinners or previous cesarean section and the significance remained, see “Table 3”. Then we performed a sensitivity analysis investigating a multivariate logistic regression for nulliparous women excluding the effect of previous adverse events with prematurity or cesarean section. Of 149 pregnancies included in the total cohort we included 72 pregnancies in the sensitivity analysis. While adjusting for age and either BMI or the use of beta blockers the significance remained. However, when we adjusted for age and the use of blood thinners there was no difference in risk of cesarean section in women with mWHO class >III compared with mWHO I-III.

**Table 3:** Premature delivery and cesarean section.

Prematurity	OR	Confidence interval	P-value
Unadjusted	6.7	2.6–17.4	<0.001
Adjusted for age	6.7	2.6–17.4	<0.001
Adjusted for age, BMI	5.2	1.9–14.6	0.001
Adjusted for age, use of blood thinner	5.8	2.1–15.5	<0.001
Adjusted for age, use of betablockers	5.6	2.1–15.0	<0.001
Adjusted for age, previous cesarean section	6.1	2.3–16.1	<0.001
**Cesarean (all, n = 149)**			
Unadjusted	5.1	2.0–12.6	<0.001
Adjusted for age	5.3	2.1–13.2	<0.001
Adjusted for age, BMI	4.8	1.8–12.5	0.001
Adjusted for age, use of blood thinner	4.4	1.7–11.3	0.002
Adjusted for age, use of betablockers	4.4	1.8–11.3	0.002
Adjusted for age, previous cesarean section	4.9	1.8–13.0	0.001
**Cesarean (nulliparous, n = 72)**			
Unadjusted	6.5	1.6–26.3	0.009
Adjusted for age	6.9	1.7–28.4	0.008
Adjusted for age, BMI	7.6	1.8–32.5	0.006
Adjusted for age, use of blood thinner	3.8	0.8–17.9	0.097
Adjusted for age, use of betablockers	5.6	1.3–24.1	0.021

Univariate and multivariate logistic regression analysis with mWHO (mWHO group I-III and mWHO group III-IV) as independent variable and premature delivery and cesarean section as dependent variable, respectively. The logistic regression for cesarean section is subdivided into an analysis with all pregnancies and an analysis with only nulliparous women. Prematurity is defined as giving birth prior to 37+0 weeks of gestational age. mWHO = modified World Health Organization class. OR = Odds Ratio.

**Fig 2:**
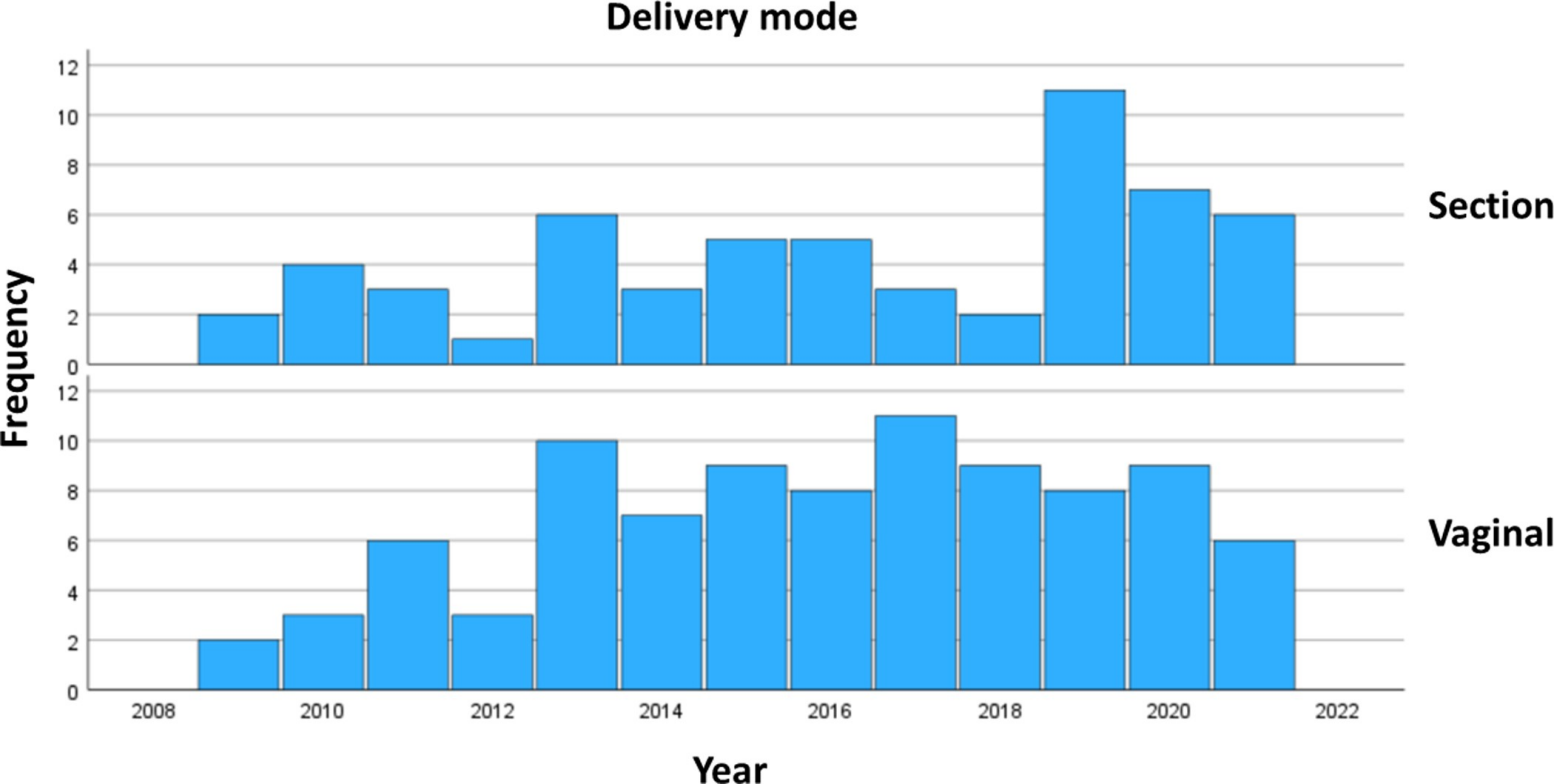
Delivery mode according to year in women with adult congenital heart disease.

Delivery mode according to year is displayed in (Fig 2). There was no increased rate of cesarean section over time (the Cochran-Armitage χ2, p = .6018) or changes in WHO groups over time (the Cochran-Armitage χ2, p = .2247). “Table 2” also displays cardiac or non-cardiac indication for induction and elective cesarean section. Out of the emergency cesarean sections, two out of 26 (7.7%) had a cardiac indication. Moreover, ten (31.3%) of the elective cesarean sections were performed on maternal request.

### Neonatal outcome

Four pregnancies ended in intrauterine fetal death and in one pregnancy the woman was induced due to a serious cerebral neonatal malformation (Fig 1). These five pregnancies were excluded in the analysis. In two of the intrauterine fetal deaths the woman had a low saturation during pregnancy. At birth, two neonates had a ventricular septal defect, two had an ASD while one neonate had both a ventricular septal defect and an ASD, see “Table 4”.

**Table 4:** Fetal/neonatal outcome. Data are shown as numbers and as percentage. Normally distributed continuous variables are presented as mean ± SD or proportions. Non-normally distributed variables are presented in non-logarithmic format as median and interquartile range (IQR). Fisher’s exact test was used to test for statistical significance between categorical variables and mWHO groups. mWHO class = modified World Health Organization class. HT = hypertension. HELLP syndrome = Hemolysis, Elevated Liver enzymes and Low Platelet syndrome. FGR = fetal growth restriction. SGA = small for gestational age.

	mWHO I (%) n = 36 (24.1%)	mWHO II (%) n = 43 (28.9%)	mWHO II-III (%) n = 43 (28.9%)	mWHO III (%) n = 24 (16.1%)	mWHO IV (%) n = 3 (2.0%)	Mis-sing (%)	*p*- value
FGR	1 (2.8)	0	0	4 (16.7)	0	0	0.007
SGA	2 (5.6)	1 (2.3)	1 (2.3)	1 (4.2)	0	0	0.831
pH umbilical artery Mean ± SD	7.2 ± 0.1	7.3 ± 0.1	7.2 ± 0.1	7.2 ± 0.1		78 (52.3)	0.801*
pH umbilical vein Median IQR	7.3 7.3–7.4	7.3 7.3–7.4	7.3 7.3–7.3	7.3 7.3–7.3	7.3	65 (43.6)	0.435*
Cardiac birth defect	1 (2.8)	1 (2.3)	1 (2.3)	2 (8.3)	0	0	0.596

Premature delivery occurred in 25 women (16.8%); 13 out of 122 women (10.7%) with an mWHO group I-III compared with 12 out of 27 (44.4%) in mWHO group III-IV. Correlations were observed between premature delivery and mWHO group (r = 0.348, p-value <0.001), delivery mode (r = 0.415, p<0.001), the use of betablockers (r = 0.274, p<0.001), blood thinners (r = 0.192, p = 0.019) and previous cesarean sections (r = 0.172, p = 0.036). The odds of premature delivery were significantly higher among pregnant women with mWHO class ≥III (OR, 6.7; 95% CI, 2.6–17.4; *p*<0.001). Then we performed a stepwise multivariate logistic regression analysis adjusting for age, the use of betablockers, blood thinners, previous cesarean sections and the significance remained, see “Table 3”.

“Regarding prematurity < 37 weeks, nine (47.4%) were iatrogenic (planned cesarean sections) compared to 10 (52.6%) spontaneous deliveries. Regarding prematurity week 28–32, none were iatrogenic and six (100%) were spontaneous deliveries. We had no extreme prematurity (<28 weeks). We noted no difference between mWHO groups (p = 0.831) in prevalence of SGA, with 5.6% SGA in mWHO class I, 2.3% in mWHO class II, 2.3% in mWHO class II-III, 4.2% in mWHO class III and no neonates with SGA in mWHO class IV.

## Discussion

This study indicates that women with more complex heart disease (mWHO classes III or IV) tend to have neonates affected by FGR, give birth prematurely and deliver by cesarean section more often than women with less complex heart disease. Previously, obstetric and neonatal complications have been described in northern and western Sweden but FGR have not been described in the Swedish pregnant ACHD population before [12, 13]. FGR is an interesting outcome due to the association between reduced cardiac function and placental insufficiency [14, 15]. Also, there seems to be national differences in management of women with ACHD in Sweden. For instance, in northern and western Sweden all women with ACHD are discussed at the tertiary center while some of the ACHD women with less complex heart disease in southern Sweden are discussed and managed at a large nearby hospital and not at our tertiary center. Thus, we sought to investigate perinatal outcome (including FGR) in our cohort in order to better stratify for risk and improve preconceptional counselling as well as pregnancy management in ACHD women in this setting.

In a cohort in western and northern Sweden, Furenäs et al. reported a preterm birth rate of 8% compared to our preterm rate of 16.8%. However, this might reside in a difference in ACHD complexity between our two Swedish cohorts, especially since the study by Furenäs et al. included 40.3% women in mWHO class I (study years 1997–2015), compared to the 24.2% of women in mWHO class I in our study (study years 2009–2021). The data is from two different centers but might suggest a trend of increased rate of women with complex congenital heart disease giving birth over time [12]. Another reason could be that less complex heart defects might be managed at the nearby secondary center University Hospital in Malmö and therefore not discussed at the multidisciplinary conferences at the tertiary ACHD center at Lund University Hospital. We demonstrated a difference in iatrogenic prematurity, where women in higher mWHO classes were more likely for this outcome. Accordingly, studies in Denmark (Kloster et al.) and New Zealand (Kirby et al.) demonstrated a higher risk of preterm birth in women with more complex congenital heart disease [9, 16]. According to the literature, the preterm birth rate is 8.0–17.1% in ACHD women [12, 13, 16–20].

In our study 16.8% of all deliveries were premature and, of these, 4.0% of the neonates were delivered before week 32 and no neonates before week 28. Comparingly, in the general population of Sweden in 2016, 5.6% of all deliveries were premature and, out of these, 0.3% of the premature deliveries occurred before week 28 and 0.6% before week 32 [21].

Additionally, we found that women in a higher mWHO class were more likely to be delivered by cesarean section than through vaginal delivery, similarly to the results of a study by Hrycyk and colleagues [22]. We found an overall rate of cesarean section of 38.9%, which is higher compared to previous reported rates of cesarean section in women with ACHD in the northern and western Sweden [13]. The frequency of cesarean section increases with a more complex mWHO class since those women more often have an indication for elective cesarean section [4]. However, it is worth noting that a proportion of this impact could be attributed to prior cesarean sections although we still note significantly higher odds for cesarean section while adjusting for age and previous cesarean section. The presences of previous cesarean deliveries exhibited the highest correlation with mode of delivery. If a previous cesarean section was recommended based on the mWHO class, the same recommendation is likely to be made in subsequent pregnancies. Furthermore, to investigate the odds of cesarean section without the effect of previous cesarean sections we performed a sensitivity analysis on nulliparous women. Still, we noted significantly higher odds for cesarean section while adjusting for age, age and usage of betablockers respectively age and BMI. While adjusting for age and blood thinner we did not note significantly higher odds. However, the confidence intervals are wide and we only included 72 pregnancies of the total 149 pregnancies.

According to guidelines from the European Society of Cardiology women in mWHO classes III and IV should deliver at an expert center for pregnancy and cardiac disease [4]. Thus, need for timing regarding competence and preparedness might be key. Roos-Hesselink et al. studied prospective pregnancies in more than 50 countries in the Registry of Pregnancy and Cardiac disease (ROPAC) and found a cesarean section rate of 44%. However, this study also included women with cardiomyopathy or ischemic heart disease [11]. In our study a total of 38.9% delivered by cesarean section compared to 19–46.6% according to literature [13, 16, 17, 19, 22]. In comparison, according to The Swedish Pregnancy Register, approximately 20% of all women delivering in 2021 at either Lund University Hospital or Malmö University Hospital delivered by cesarean section [23]. Out of the elective cesarean sections, 31.3% were due to maternal request possibly indicating psychological reasons such as maternal anxiety concerning delivery. In a previous study investigating self-reported quality of life in ACHD women in Skåne County, we noted a significantly higher rate of cesarean section in women who described a higher rate of anxiety/depression before pregnancy [24]. Planned cesarean section might be perceived as safer than vaginal birth by medical doctors, although cesarean section is associated with more complications and is not recommended for women with heart disease other than for obstetric indications or cardiac conditions with a very high risk. ROPAC data showed that there were no maternal benefits of delivering by an elective cesarean section. Rather, cesarean section results in more infections, more venous thromboembolisms and more blood loss [4].

Of our ACHD women, 7% bled >1000 ml postpartum. In comparison, Furenäs et al. reported an incidence of 8–10% for postpartum bleeding [12, 13]. We did not note any significant differences in rate of hemorrhage although we noted a difference in the use of blood thinners. According to The Swedish Pregnancy Register; 7% with vaginal delivery and 15% with cesarean section bled more than 1000 ml of all deliveries in Sweden in 2021 [23]. According to the literature, postpartum hemorrhage complicates 0.9–25.8% of ACHD deliveries; [16, 18, 20, 22] however, different diagnostic criteria of postpartum hemorrhage may make comparisons difficult.

Fetal growth is often measured and presented in the literature by the outcome SGA [13, 19, 25]. Although, the SGA group becomes physiologically heterogeneous when FGR is not distinguished. As previously described in the methods section, SGA is a measurement of low birth/fetal weight while FGR is a measurement of failure to reach the intrauterine growth potential. Studies have revealed FGR to be an interesting outcome due to the demonstrated association between placental insufficiency and reduced cardiac performance [14, 15]. Regarding FGR, we found an incidence of 3.4% and in comparison, Hayward et al. reported an incidence of 2.6%. We found higher incidence in women in mWHO classes III and IV compared to those in mWHO classes I, II and II-III “Table 4”. Similarly, Hayward et al. reported a higher incidence of FGR among women with complex congenital heart disease (5.3%) compared to those with a non-complex condition (2.4%) [17]. Owens et al. reported an incidence of 1% of FGR in ACHD women [18]. However, we also found a significant difference in the use of betablockers, where women with more complex mWHO classes were more prone to medicate with betablockers. It is known that beta blockers may increase the risk of FGR [4]. Further, larger studies are warranted to confirm if FGR is indeed more common in women with more complex ACHD, or if this is due to an increased use of beta blockers in these women.

Ramage and colleagues found that women with ACHD have higher odds for giving birth to a neonate with SGA compared to women without ACHD, although this did not apply to all ACHD subgroups [19]. We did not find any difference in the incidence of SGA in relation to the mWHO class. However, we did not use the same definition of SGA as Ramage et al. Furenäs et al. used the same definition of SGA -2SD compared to the Swedish average for gestational age and gender. Furenäs et al. also report a similar incidence compared to our study [13].

We found that 3.4% of the neonates were born with a cardiac birth defect. However, the national incidence in Sweden is 1% [2]. Accordingly, Lammers et al. found that women with ACHD were 6.1 times more likely to give birth to a child with cardiac birth defect [26]. In comparison, the literature reports an incidence of 2.9–7.0% [13, 16, 18].

The retrospective study design is a drawback and might reside in a selection bias. We could only include patients discussed at multidisciplinary conferences, and there might be some patients with less severe ACHD omitted since these women had no contact with the ACHD center during pregnancy and delivery. Furthermore, some women with the most severe ACHD are advised against pregnancy and some of these women with severe ACHD might be unable to obtain a pregnancy. Another limitation is the relatively small number of women included and the heterogeneity of included diagnoses; thus, we made analyses based on mWHO class and not individual diagnoses. Lastly, previous cesarean section correlates with the risk of having another cesarean section at a following pregnancy.

## Conclusion

Our findings indicate that women with more complex heart disease (mWHO classes III or IV) tend to have neonates affected by FGR, give birth prematurely and deliver by cesarean section more often than women with less complex heart disease. Regarding other neonatal and obstetric complications, we found no other differences in relation to mWHO class; however, our study population was relatively small and further, larger and ideally prospective studies are warranted.

## Supporting information

S1 Table Pain relief in pregnant women with congenital heart disease. (DOCX)

## Abbreviations

ACHD: Adult Congenital Heart Disease
ASD: atrial septal defect
BFC: blood flow class
DORV: double outlet right ventricle
ECG: electrocardiogram
FGR: fetal growth restriction
IQR: interquartile range
mWHO: modified World Health Organization
SGA: small for gestational age
SWEDCON: Swedish Registry of Congenital Heart Disease
VBAC: vaginal birth after cesarean section
