# Supplementary material for: Increased cesarean section rate and premature birth according to modified WHO maternal cardiovascular risk in pregnant women with congenital heart disease
Source: PLoS One. 2023 Nov 16;18(11):e0294323. doi: 10.1371/journal.pone.0294323 (PMC10653484; doi:10.1371/journal.pone.0294323)
Supplement: S1 Table — (DOCX) [file pone.0294323.s001.docx]

**S1 Table.** Pain relief in pregnant women with congenital heart disease.

TENS = Transcutaneous electrical nerve stimulation.

|  | mWHO I  (%)  n=36 (24.1%) | mWHO II (%)  n=43 (28.9%) | mWHO II-III (%)  n=43 (28.9%) | mWHO III (%)  n=24 (16.1%) | mWHO IV (%)  n=3  (2.0%) | Mis-  sing  (%) | |  |
| --- | --- | --- | --- | --- | --- | --- | --- | --- |
| Pain relief  Epidural anesthesia  Spinal anesthesia  Nitrous oxide /oxygen  General anesthesia  Pethidine/  morphine  Pudendal anesthesia  Bath  TENS  Infiltration  Acupuncture  Other  None | 12 (33.3)  7 (19.4)  26 (72.2)  1 (2.8)  8 (22.2)  0  0  1 (2.8)  6 (16.7)  1 (2.8)  0  0 | 7 (16.3)  11 (25.6)  23 (53.5)  2 (4.7)  9 (20.9)  1 (2.3)  1 (2.3)  1 (2.3)  1 (2.3)  1 (2.3)  5 (11.6)  1 (2.3) | 9 (20.9)  9 (20.9)  26 (60.5)  2 (4.7)  5 (11.6)  0  2 (4.7)  2 (4.7)  8 (18.6)  0  1 (2.3)  1 (2.3) | 1 (4.2)  7 (29.2)  7 (29.2)  10 (41.7)  1 (4.2)  0  1 (4.2)  1 (4.2)  2 (8.3)  0  1 (4.2)  0 | 0  1 (33.3)  0  2 (66.7)  0  0  0  0  0  0  0  0 | | 3 (2.0) | |
